# Progress in Disease of Digestive Organs

**Published:** 1903-11-21

**Authors:** 


### Progress in Disease of Digestive Organs.

**Mastication.**—The importance of efficient mastication is insisted upon by Campbell[1] both as an integral part of digestion and as an aid to the proper development of the jaws, teeth, and nasopharynx. Its first function consists in finely dividing the food so that it is easily acted upon by the gastric juices, and so called indigestible foods are well borne by many dyspeptics if thoroughly masticated. It also reflexly promotes the flow of saliva and thus stimulates the stomach. Its effect on development is hardly less important; the blood supply and nutrition of the parts are improved, and hence narrow jaws, dental caries, pyorrhœa alveolaris, and adenoid vegetations are avoided. The present age is one of soft foods, which decrease the necessity for mastication and are thus responsible for many modern ailments—appendicitis and adenoids being among the most prominent.

**Gastric Ulcer.**—Half the cases of gastric ulcer are cured by medical means, and 1 per cent. of all gastric cancers originate in ulcer (Cordier[2]). The medical treatment is physiological rest. Rectal feeding for a week to a fortnight with not even water by the mouth is advocated by Rolleston, Collier, Boyd, Griffiths, Dawson[3]; King would allow water, and Saundby considers that only in case of hæmatemesis should food be withheld, and then only for 48 hours. Collier, Boyd, and Dawson consider the actual nutritional value of enemata very slight. Washing out of the rectum daily (Rolleston) or every third day (Griffiths) should be ordered. Bleeding and perforation are the indications for operation, but bleeding often comes from multiple points or minute openings (McPhedran, Dawson), and if alarming is a contraindication for operation (Cordier). Adrenalin is recommended by Rolleston, though King has employed it without effect, and Dawson considers it and ergot dangerous; but Rolleston agrees with Parker in advising calcium chloride. The operation of gastroenterostomy is advocated by Cordier, though Boyd considers it useless. Operative interference is only justified by persistent pain, recurrent hæmorrhages, and obstruction. The pain may be due to adhesions (McPhedran). Rodgers[4] has tabulated six cases of gastric and duodenal ulcer to show the uselessness of the obliteration of liver dulness as a sign of perforation. It appears, however, to bear some relation to the contents of the stomach. The dulness is least marked during rectal feeding, and more marked after a meal than before it. The occurrence of vomiting of a reflex nature during rectal feeding has been demonstrated by Rolleston and Jex Blake[5] by an analysis of 171 cases of gastric ulcer at St. George's. Twenty-seven per cent. of all cases so fed vomit, but in the rather larger proportion this is due to oral sepsis.

**Hyperchlorhydria.**—This condition, which is associated with many diseases, such as mucous colitis (Bottentuit) and some cases of gastric ulcer (Rolleston), is not uncommon in carcinoma, but, according to Gwyn,[6] extreme degrees in this disease are rare. He quotes a case in which the total acidity was 70, and free hydrochloric acid up to 55 was found four months after onset. Later on it diminished and attacks of stupor of obscure origin supervened. Meunier[7] considers that hyperchlorhydria should only be diagnosed when, after Ewald's diet, the gastric juice contains saccharine matter of reducing power less than 1 per cent. dextrose, or soluble starch derivatives yielding less than 2 per cent. dextrose. A method of estimating clinically the amount of HCl. in gastric contents has been devised by Ham and Macleod.[8] It only requires an ordinary polarimeter and gives practically the same results as Hoffmann's, on which it is based. Dextrorotary solutions of approximately the same strength are used, and all are "incubated" for exactly the same time. In one is a known amount of acid, in the other the gastric juice to be tested, together with grape sugar. The amount of acid in the unknown solution will be obtained by multiplying the number of degress of inversion it produces by the amount of acid in the known solution and dividing the product by the degrees of inversion produced by the latter.

**Gastric Tetany** has been described as a rare and almost necessarily fatal condition, but Moynihan,[9] who has recently described a severe case thinks that slight attacks are not uncommon, but that they are unnoticed till they culminate in a perhaps fatal manner. It is thus a preventible condition if taken early and the treatment is gastro-enterostomy.

**Hiccough** is certainly another condition frequently seen in its slighter phases; a bad case has been described by Atlee.[10] It occurred in an old man who after three days of intermittent attacks hiccoughed continuously for four days. A calomel purge, counter irritation to the epigastrium and a mixture of bismuth and aromatics quieted the spasms, but they soon returned and were finally overcome by frequent doses of chloral. None of the ordinary causes—reflex or mechanical irritation, central disease, or toxic absorption could be proved to have operated in this case.

[1] Lancet, 1903, ii., pp. 84, 150, 216, 375. [2] Med. Rec., 1903, i., p. 755. [3] Ibid., ii., p. 271. [4] Lancet, 1903, ii., p. 98. [5] Brit. Med. Jour., 1903, ii., p. 68. [6] Med. Rec., 1903, i., p. 817. [7] Ibid., ii., p. 268. [8] Lancet, 1903, ii., p. 313. [9] Bact., March, 1903. [10] Amer. Jour. Med. Sci., July, 1903.

*(To be continued.)*

